# Intestinal Immune System and Amplification of Mouse Mammary Tumor Virus

**DOI:** 10.3389/fcimb.2021.807462

**Published:** 2022-01-13

**Authors:** Lankai Chen, Xipeng Zhang, Guisheng Liu, Shuo Chen, Minying Zheng, Siwei Zhu, Shiwu Zhang

**Affiliations:** ^1^ Nankai University School of Medicine, Nankai University, Tianjin, China; ^2^ Department of Colorectal Surgery, Tianjin Union Medical Center, Tianjin, China; ^3^ Department of Pathology, Tianjin Union Medical Center, Tianjin, China

**Keywords:** mouse mammary tumor virus, superantigen, antigen-presenting cells, T cell, Peyer’s patch

## Abstract

Mouse mammary tumor virus (MMTV) is a virus that induces breast cancer in mice. During lactation, MMTV can transmit from mother to offspring through milk, and Peyer’s patches (PPs) in mouse intestine are the first and specific target organ. MMTV can be transported into PPs by microfold cells and then activate antigen-presenting cells (APCs) by directly binding with Toll-like receptors (TLRs) whereas infect them through mouse transferrin receptor 1 (mTfR1). After being endocytosed, MMTV is reversely transcribed and the cDNA inserts into the host genome. Superantigen (SAg) expressed by provirus is presented by APCs to cognate CD4^+^ T cells *via* MHCII molecules to induce SAg response, which leads to substantial proliferation and recruitment of related immune cells. Both APCs and T cells can be infected by MMTV and these extensively proliferated lymphocytes and recruited dendritic cells act as hotbeds for viral replication and amplification. In this case, intestinal lymphatic tissues can actually become the source of infection for the transmission of MMTV *in vivo*, which results in mammary gland infection by MMTV and eventually lead to the occurrence of breast cancer.

## Introduction

Mouse mammary tumor virus (MMTV) is first reported by Bittner in his mouse adoptive nursing experiment (he named the virus “milk factor”) which was proved to be associated with carcinogenesis between progeny and their breeders (Bittner, 1936). The subsequent *in vivo* inoculation experiments of viral particles isolated from mouse milk as well as the advances in experience and techniques for purifying RNA viruses finally confirmed that MMTV is a single-stranded type B complex retrovirus (Graff et al., 1949; Dmochowski and Grey, 1957; Duesberg and Blair, 1966). Endogenous mouse mammary tumor virus (Mtv) exists in genomes of multiple mouse strains and is passed on to offspring through germ lines. Most Mtv strains are defective of producing infectious viral particles but retain the ability to encode functional superantigen (SAg). The negative selection induced by SAg during thymus development deletes cognate thymocytes bearing Vβ chains thus shapes the T cell repertoire (Salmons et al., 1986; Woodland et al., 1991; Cho et al., 1995).

In general, the immune system plays an important role to fight against foreign pathogens. However, some viruses are competent to disrupt the immune system. The most classic example is human immunodeficiency viruses (HIV), which induces low levels of CD4^+^ T cells and eventually lead to fatal conditions including severe infections and malignant tumors (Moir et al., 2011). Both MMTV and HIV can infect immune cells and MMTV infection cannot ruin the immune system. Instead, MMTV can depend on the immune system for amplification. SAg expressed by MMTV provirus plays an important role in this process. SAg can be presented to cognate CD4^+^ T cells by antigen-presenting cells (APCs) and induces immune response which causes extensive proliferation of lymphocytes. MMTV replicates and expands in infected immune cell populations while evading immune killing effect. MMTV-infected lymphocytes migrate to mammary glands where the virus can infect and induce breast cancer by activating pro-oncogenes near the integration site. During the process of MMTV infection, SAg encoded by MMTV can induce the deletion of specific Vβ-bearing T cells. MMTV SAg can also lead to the deletion of cognate Vβ CD4^+^ T cells in chronic infection. In this review, we will focus on illustrating the relationship between MMTV life cycle and the intestinal immune system resting on existing evidence and put forward some unsolved problems to encourage further studies about relationships between MMTV and breast cancer.

## MMTV Enters Peyer’s Patches (PPs) to Preactivate and Infect B Cells

MMTV viruses can be found in the milk secreted by MMTV-infected mothers (Miroff and Magdoff-Fairchild, 1965; Duesberg and Blair, 1966; Hainaut et al., 1983) and are ingested by suckling neonates during breastfeeding. Hainaut et al. proposed that the stomach could not be the portal of entry for MMTV and viral antigen including main envelope glycoprotein gp52 and main core protein p28 could not be detected in the stomach walls (Hainaut et al., 1983). After ingestion of milk, some MMTV viral particles were retained in the stomach for a long time and the remainder reached the small intestine (Hainaut et al., 1983). Viral antigen detection and electron microscopy observation suggest that viral particles are progressively digested and denatured during intestinal transit. Some of the viral particles appear to penetrate intestinal epithelial cells by Peyer’s patches (PPs) (Hainaut et al., 1983; Bevilacqua and Marchetti, 1985). PPs are composed of lymphatic follicles in the small intestinal mucosa of human and mouse and are important component of the intestinal mucosal immune system (De Jesus et al., 2013). Karapetian et al. (1994) demonstrated that PPs were the specific targets for early infection by using semiquantitative PCR assay. MMTV (SW: detected in the milk of V_β_6-deleting BALB/c IC mice) (Held et al., 1992) DNA was exclusively detected in the PPs of neonatal mice at day 4 and 9, whereas the secondarily infected site was the mesenteric lymph nodes (with viral DNA at day 14). Microfold cells (M cells) in the follicle-associated epithelium of newborn mice may help transport MMTV from the intestinal lumen to the basolateral side of PPs *via* fluid phase endocytosis (Gonnella and Neutra, 1984; Bevilacqua et al., 1989; Golovkina et al., 1999).

By injecting MMTV (SW) into the footpads of BALB/c mice, Ardavin et al. (1997) demonstrated that ~80% of B cells were transiently activated in the popliteal lymph nodes at ~18 h without cognate T cell activation. Subsequently, the percentage of activated B cells declined to normal levels (before 29 h). This early activation of B cells may be associated with MMTV (SW) because retroviral cDNA was found exclusively in activated CD69^+^ B cells. Moreover, B cells in draining lymph nodes increased by three- to five-fold by recruitment and/or retention. The bromodeoxyuridine incorporation assay and cell cycle analysis proved that cell division was not the cause of the B cells amplification (Ardavin et al., 1997). However, neither β7 integrins nor L-selectin (CD62L) were indispensable for lymphocyte recruitment during MMTV infection (Czarneski et al., 2002), suggesting that some other lymphocyte homing receptors may be involved in this process. The significance of B cell preactivation during MMTV infection is unclear. Results of Finke et al. (1998) showed that naive small resting B cells, but not pre-activated B cells, were the major targets for MMTV infection. In other words, MMTV efficiently infected B cells without preactivation. It is well known most retroviruses can only integrate their cDNA into host chromosomes during cell division (Roe et al., 1993; Suzuki and Craigie, 2007). As a retrovirus, the competence of MMTV for infecting quiescent B cells is similar to HIV activity in monocytes (Weinberg et al., 1991). This similarity and potential mechanism is worthy of further study. Information regarding the receptors through which MMTV activates and/or infects target cells is presented in **Box 1**.

Box 1 **Receptors used by MMTV to enter or activate target cells.**

Through phenotypic screening of T31 mouse/hamster radiation hybrid panel, Ross et al. (Ross et al., 2002; Ross, 2010) demonstrated that mouse transferrin receptor 1 (mTfR1, CD71) was the receptor for MMTV infection by binding with Env protein. The virus was endocytosed *via* clathrin-coated pits and the acidic endosomes were trafficked to an acidic compartment to induce Env-plasma membrane fusion. Moreover, human cells bearing mTfR1 (not human TfR1) could also be susceptible to infection by virus bearing MMTV Env (Ross et al., 2002; Courreges et al., 2007). We infer that cells expressing mTfR1 can be infected by MMTV. Correspondingly, studies shown that only TfR1 from mice and rats could function as entry receptors for MMTV but not those from cats, dogs, hamsters, or humans. This function is independent of the host’s iron status (Ross et al., 2002; Wang et al., 2006).

Toll-like receptor 4 (TLR4) has been shown to be associated with the activation of B and dendritic cells (DCs) through binding MMTV Env protein, and this direct interaction is independent of N-linked glycosylation of the Env protein (Golovkina et al., 1998; Rassa et al., 2002; Burzyn et al., 2004). This activation process was likewise independent of MMTV expression (Rassa et al., 2002; Burzyn et al., 2004). The positive correlation between TLR4 expression and B cell class switching also provided a lateral demonstration (Cabrera et al., 2010). However, whether TLR4 is essential for MMTV-related activation or not and the exact significance of TLR4 in the infectious process of MMTV remain unclear. In addition, TLR2 could participate in the maturation of MMTV-induced bone marrow-derived dendritic cells (BMDCs) (Burzyn et al., 2004). MMTV directly binds to different receptors in order to activate or enter target cells. Only a very small proportion of early activated B cells could be infected by MMTV (Ardavin et al., 1997) and viral infection was independent of the preactivation of target cells (Finke et al., 1998).

## MMTV-Infected APCs Present SAg by MHCII Molecule

B cells in PPs were essential for the MMTV life cycle (Beutner et al., 1994) and several pieces of evidence supported B cells as the primary targets of MMTV (Held et al., 1993; Held et al., 1993; Beutner et al., 1994; Karapetian et al., 1994). It was reported that DCs rather than B cells may be the first targets of MMTV infection. Courreges et al. (2007) demonstrated that DCs were the first cells to be infected by MMTV *in vivo*. Researchers injected MMTV Env-pseudotyped recombinant murine leukemia virus tagged with the GFP gene into the footpad of naive mice and lymphocytes were collected from draining lymph nodes for fluorescence activated cell sorting (FACS) analysis 24 h after injection. The results showed that only DCs (CD11c^+^) were GFP positive.

**Figure 1:**
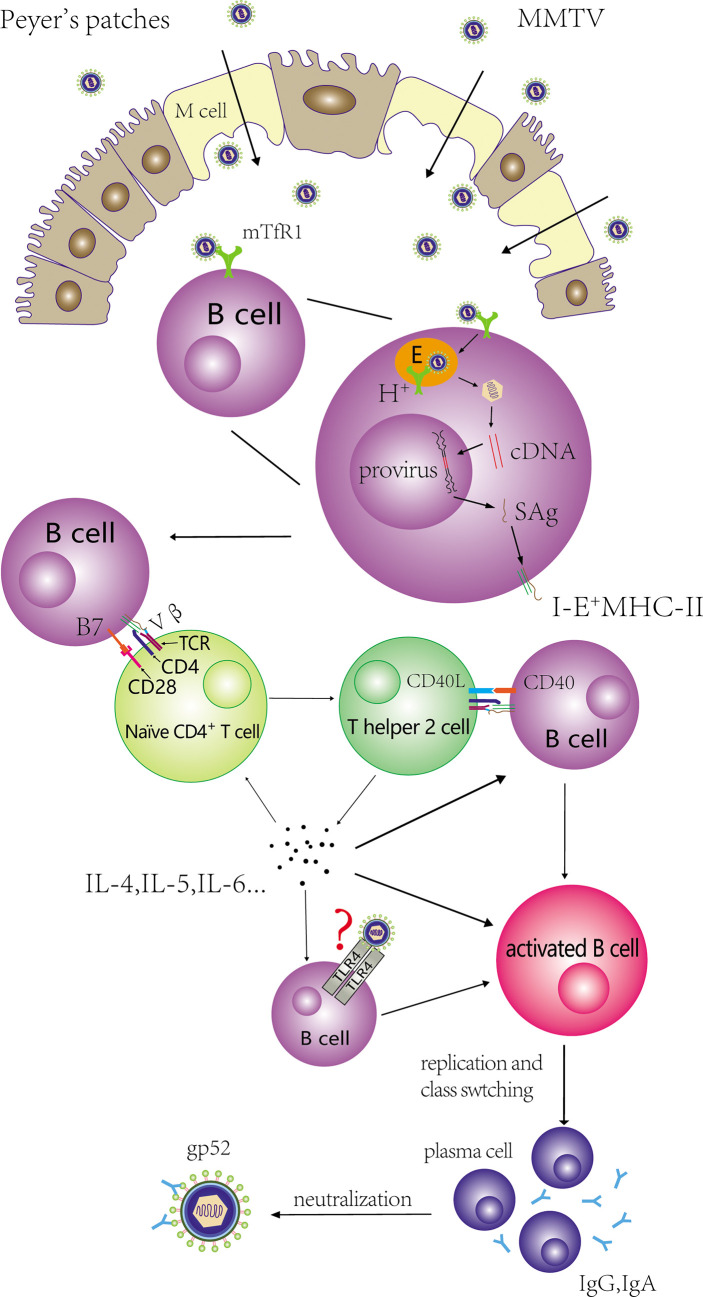
Activation and proliferation of CD4+ T cells and B cells mediated by MMTV. MMTV is transported from intestinal lumen into PPs with the help of M cells, and binds to mTfR1 on B cells. The virus is endocytosed *via* clathrin-coated pits and the acidic endosomes are trafficked to an acidic compartment to induce Env-plasma membrane fusion. MMTV infect these cells and integrate the provirus into the genome of B cells. SAg expressed by MMTV provirus is presented by I-E^+^ MHCII molecules, and binds to the Vβ domain of TCR. In addition, CD28-B7 interaction also contributes to the activation of naïve CD4^+^ T cells. The activated CD4^+^ T cells can differentiate into Th2 cells, and Th2 cells can stimulate extensive B cells. The process involves the interaction of CD40L-CD40. Activated B cells can proliferate and produce antibodies that target MMTV GP52 for neutralization.

DCs could effectively present MMTV SAg to T cells (Ardavín et al., 1996; Martín et al., 2002; Courreges et al., 2007). B cells were used as an example to illustrate the details of SAg presentation. The mature MMTV virion did not contain SAg transcripts and protein (Smith et al., 1987; Held et al., 1993). B cells express SAg *via* the regulation of proviral transcriptional enhancers. Promoters and enhancers within MMTV 5′LTR are not needed for transcription. A 183-bp region in the RNase H domain of the *pol* gene and an intragenic enhancer within the *sag* gene itself were essential for the expression of SAg in B cells and pro-B cells, respectively (Reuss and Coffin, 1995; Reuss and Coffin, 1998). MMTV infect B cells and integrate the provirus into the genome of B cells. SAg expressed by MMTV provirus is presented by I-E^+^ MHCII molecules. The expressed SAg protein combined with MHCII molecules to form complexes on the surface of B cells, and then bound to the Vβ domain of T cell receptor (TCR) expressed by corresponding CD4^+^ T cells (Held et al., 1993; Acha-Orbea and MacDonald, 1995). SAg bound outside the antigen groove of MHCII molecules (Dellabona et al., 1990) (**Figure 1**). Fortin et al. proposed a model presenting SAg/MHCII/TCR interactive architecture (Fortin et al., 2014). In this model, SAg cross-linked two MHCIIs by noncovalent N- and C-terminal moieties, and TCRβ CDR3 was skewed to bind the MHCII α-chain instead of the antigenic peptide. The glycosylated extracellular C-terminal region of SAg determined the specificity of interacting TCR Vβ chain and the subsequent activation of corresponding T cells (Held et al., 1992; Held et al., 1993; Yazdanbakhsh et al., 1993; Astori and Karapetian, 1998). Hence, different MMTVs had diverse specificity toward TCR Vβs. Vβ6^+^ CD4^+^ T cells interacted exclusively with MMTV(SW) SAg (Held et al., 1993; Ardavin et al., 1997) and MMTV(C3H, a kind mouse strain with spontaneous breast cancer) only influenced the activation of CD4^+^ T cells bearing Vβ14^+^ and Vβ15^+^ TCRs (Choi et al., 1991; Held et al., 1993). SAg encoded by MMTV (II-TES14, a mouse mammary tumor virus encoding a superantigen specific for TCR Vβ14) and MMTV (II-TES2, a mouse mammary tumor virus encoding a superantigen specific for TCR Vβ2) was specific for Vβ14^+^ and Vβ2^+^ TCR (Ando et al., 1995; Upragarin et al., 1997). Based on studies determining exact regions of Vβ chains that interacted with endogenous SAg, the Mls-1^a^ first complementarity-determining region and some residues on the lateral solvent-exposed β-pleated sheet in Vβs were associated with SAg activity (Pullen et al., 1990; Pullen et al., 1991).

## Activation and Proliferation of CD4^+^ T Cells and B Cells

Experiments demonstrated that B cells and SAg-reactive CD4^+^ T cells in draining lymph nodes were activated and proliferated over the course of 1-3 days after MMTV (SW) injection to the hind footpad of 6-10-week-old female mice. The SAg response reached its peak on approximately day 6 (Held et al., 1993; Ardavin et al., 1997; Rassa et al., 2002). The time course of these events was postponed in neonatal mice nursed by MMTV (SW)-positive mothers. The SAg response could first be detected in PPs on approximately day 5 after birth and reach its climax at approximately 6-9 days (Karapetian et al., 1994). The delay may be ascribed to: (a) the process of MMTV ingestion, reaching the gut, and transportation *via* M cells in order to reach PPs; (b) virus titers differing between methods of infection; (c) the composition of lymphocyte populations at the initial stage varying from draining lymph nodes to PPs; and (d) other interfering factors that vary among laboratories, including experimental instruments, viral activity, reagents, and operational skills.

Compared with CD4^+^ T cells, CD8^+^ T cells did not show a significant activation and increase in draining lymph nodes after injection of MMTV (SW) (Ardavin et al., 1997; Acha-Orbea et al., 1999). This finding may be attributed to CD8^+^ T cells lacking the ability to bind to the SAg/MHCII complex. The inadequacy of SAg-presenting cells may account for this phenomenon since Mtv-congenic splenocytes which can express endogenous SAg in all APCs. Portional expression of SAg from MMTV infection were able to activate and caused an increase in CD8^+^ T cells bearing corresponding Vβ elements in lymphatics (Webb and Sprent, 1990) and spleens after intravenous injection (MacDonald et al., 1990). The process seemed to depend on help from CD4^+^ T cells, and APCs with I-E^+^ MHCII alleles were more stimulatory to CD4^+^ and CD8^+^ T cells than those of I-E^-^ (MacDonald et al., 1990; Webb and Sprent, 1990; Chvatchko and MacDonald, 1991; Chervonsky et al., 1995).

Activation of SAg-reactive CD4^+^ T cells was essential for the subsequent extensive activation and expansion of MMTV-infected B cells. Held et al. (1993) showed that cycling T cells were observed earlier than cycling B cells in the local immune response following virus injection. Moreover, both transgenic mice whose TCR Vβ chains specific to MMTV(SW)/Mtv-7 SAg were allelically excluded (TCR Vβ8.2) and BALB.D2 mice (Mtv-7+) with >95% deletion of Mls-1^a^-reacting Vβ T cells failed to induce B cells proliferation in draining lymph nodes after injecting the same MMTV(SW) (Held et al., 1993; Held et al., 1993). In addition to the binding of SAg/MHCII complexes with cognate Vβ TCR, studies have shown that CD28-B7 interaction contributed to the activation of MMTV SAg-reactive T cells (Palmer et al., 1996; Champagne et al., 1996) and CD40L-CD40 interaction was necessary for activating B cells *via* activated T cells in the MMTV life cycle (Chervonsky et al., 1995). Increases in IL-4 mRNA in draining lymph nodes after injecting MMTV(SW) into the hind footpads of adult mice indicated that activated T cells could differentiate to T-helper 2 (Th2) cells (Toellner et al., 1998). On the other hand, Th2 cells could produce IL-4 in order to induce B cell proliferation and class switching. IL-5 and IL-6 were also upregulated in these studies (Mora and Von Andrian, 2008; Cabrera et al., 2010). Jude et al. (2003) showed MMTV could stimulate macrophages and DCs through TLR4 to generate signals, which can in turn stimulate B cells to secrete IL-10. Since IL-10 can suppress immunity against retroviruses, B cells may escape the killing effects of cytotoxic T lymphocytes. In addition, Th2 can produce IL-10 which contributes to triggering Th2 cells. The induction of CD4^+^ T cells differentiated into Th2 cells and the secretion of IL-10 to evade the T lymphocyte response are beneficial to the survival and proliferation of MMTV-infected B cells, and ultimately facilitate the amplification of MMTV.

MMTV expression was not necessary for activating B cells in this phase, and not all activated B cells were MMTV DNA^+^ or mRNA^+^ (Held et al., 1993). This conclusion has been verified by other studies (Rassa et al., 2002), implying that direct Th2-B cell interaction is not necessary for B cell activation. Cytokines secreted by Th2 cells was able to activate B cells. This process requiring the activation signals from binding of MMTV to TLR4 on the surface of B cells needs to be further explored. Held et al. showed that a few of the activated B cells contained large amount of MMTV (SW) DNA. Their results showed that 19% of the presumably activated cell population contained 67% of the MMTV (SW) DNA, whereas 77% of the small resting B population contained only 11% in all B cell populations. That MMTV (SW) DNA can be detected in activated B cells suggests: (a) MMTV-infected B cells are more likely to be activated and to clonally expand, which reflects the fact that Th2-SAg presented B cell interactions play a major role in B cell activation; and (b) the activated B cells are more likely to be infected by MMTV. These results are consistent with the previous conclusion that naive small resting (but not pre-activated) B cells are major targets for MMTV infection in the early phase when pre-activated B cells did not proliferate (Ardavin et al., 1997).

After activation, class switching (CS) occurs in activated B cells, which the isotypes or subtypes of antibodies produced by activated B cells are determined. Cabrera et al. showed that both the absolute number of IgA^+^ B220^high^ and IgA^+^ B220^low^ cells increased in the PPs of pups nursed by MMTV (BALB6: isolated from BALB/cLA mice with a Vβ6^+^/Vβ8.1^+^-T-cell-specific SAg) (Golovkina et al., 1997; Cabrera et al., 2008)-infected mothers, and these researchers likewise detected CTα expression (a molecular marker of CS) by semi-quantitative PT-PCR (Cabrera et al., 2010). The expressions of IL-5 and IL-6 mRNA related to IgA CS were also upregulated (Mora and Von Andrian, 2008). Moreover, SAg-reactive T cells were indispensable for IgA^+^ cell increase (Cabrera et al., 2010), which further reinforces the reliability of the orderly occurrence of these series of activation events. Held et al. (1993) collected supernatants from lymph node cell cultures isolated from popliteal, paraaortic, and inguinal lymph nodes 3 days after *in vivo* hind-footpad MMTV(SW) injection and proved that immunoglobulin G (IgG) (especially IgG2a) was the predominant Ig response subclass. The experimental outcome indicated the necessity of SAg-reactive CD4^+^ T cells for B cell CS. The probable reasons of different immunoglobulin derived from MMTV infection may be relate with: (a) gut-associated lymphoid tissue including PPs is mucosal tissue, and B cells residing in mucosal tissue are prone to switching to IgA, whereas popliteal, paraaortic, and inguinal lymph nodes are not; and (b) different MMTV subtypes might induce different and specific CS in the SAg-related immune response. Some studies noted that antibodies produced by differentiated B cells targeted the MMTV envelope protein gp52 to exert cytotoxic and neutralizing functions (**Figure 1**) (Schochetman et al., 1979; Luther et al., 1997).

## Contribution of DCs to the SAg Response

In MMTV injection experiments, the number of DCs increased dramatically in draining lymph nodes during the first week (Martín et al., 2002; Vacheron et al., 2002; del Hoyo et al., 2002) and then decreased. The number of DCs in the PPs of pups fed by MMTV-infected mothers also increased (Burzyn et al., 2004). DCs have long been considered terminally differentiated and non-dividing in peripheral lymphoid organs. The increase is likely the consequence of massive DCs migrating into the draining lymph nodes. Further studies reveal that the recruited DCs are DC precursors (i.e., immature DCs), which are recruited from blood rather than afferent lymph and differ from B cells. The recruitment of immature DCs from blood was CD62L dependent (del Hoyo et al., 2002; Martín et al., 2002).

After recruitment, immature DCs were activated by direct binding to MMTV (Burzyn et al., 2004). Activated DCs upregulated mTfR1 and expressed some pro-inflammatory cytokines and chemokines including TNF-α, IL-6, IL-12 P40, IP-10, MIP-1α, MIP-1β, MIP-2, and RANTES (Burzyn et al., 2004). These pro-inflammatory cytokines had chemotactic effects on DC precursors and MMTV-activated DCs (Banchereau et al., 2000; Cravens and Lipsky, 2002; Courreges et al., 2007), as well as recruiting naive B and T cells to PPs (Burzyn et al., 2004). After activated by MMTV, BMDCs expressing CC chemokine receptor CCR7 increased, while BMDCs expressing CCR6 decreased (Courreges et al., 2007). DCs activated by MMTV could release cytokines, leading to the secretion of IL-10 by B cells in order to suppress antiviral immune responses (Jude et al., 2003).

Cassell (Cassell and Schwartz, 1994) demonstrated that DCs were more efficient in stimulating naive CD4^+^ T cells than B cell blasts under conditions of COOH-terminal fragment 81-104 in pigeon cytochrome c. This conclusion was consistent with a study performed by Vacheron et al. (2002) which Mtv-7-expressing APCs derived from BALB.D2 were injected into the hind footpad of congenic BALB/c mice (bone marrow-derived DCs and B220^+^ splenic B cells) and a 10- to 25-fold high efficiency for DCs priming SAg-reactive T cells in draining popliteal lymph nodes (LN) was detected compared with B cells. However, only a slight but significant proliferation of Vβ6^+^ CD4^+^ T cells was observed in a study which DCs co-cultured with T lymphocytes from BALB/c spleens *in vitro* after infection with MMTV (SW), and showed a weak capacity to induce a SAg response (Vacheron et al., 1997). MMTV-infected DCs could produce MMTV virions and affect other cells (Courreges et al., 2007) and B cells might be infected and take part in the activation of SAg-reactive T cells in draining popliteal LN. Results of another study which MMTV (SW) was injected into the footpad of B cell-deficient mice (μMT^-/-^) showed DCs alone could induce chronical peripheral deletion of superantigen-reactive T cells, and could not activate and amplify the superantigen-reactive T cells (Vacheron et al., 2002). The co-injection of endogenous SAg-expressing DCs and B cells into recipient mice resulted in the expansion of reactive T cells, suggesting an synergistic effect between DCs and B cells (Vacheron et al., 2002). This hypothesis was supported by Baribaud’s study (Baribaud et al., 1999) which showed that Vβ4 CD4^+^ T cells could be activated and expanded in transgenic mice exclusively expressing I-Eα MHCII complex on DCs after injection of MMTV(SIM, a kind of MMTV which showed reactivity with Vβ4 and Vβ10^a/c^ TCRs) into the footpad. The SAg response was inhibited by simultaneously knocking out the gene coding the IgM transmembrane region. *In vivo*, spleens are likely to contain a large number of mature DCs that migrated from nonlymphoid organs. The difference of susceptibility is associated with the role of immature and mature DCs during SAg expression and presentation. Since cytokines and other cell types may influence the observed result, experiments focused on the susceptibility difference of MMTV infection between immature and mature DCs *in vitro* may be necessary. Finally, the injection study was based on endogenous Mtv-7 (Vacheron et al., 2002), and other study was performed using exogenous MMTV(SW) (Vacheron et al., 1997). DCs from BALB/D2 mice contain two copies of Mtv-7 per cell and thereby circumvent the process of MMTV infection. The proportion of DCs expressing and presenting SAg may be different between the two conditions.

**Figure 2:**
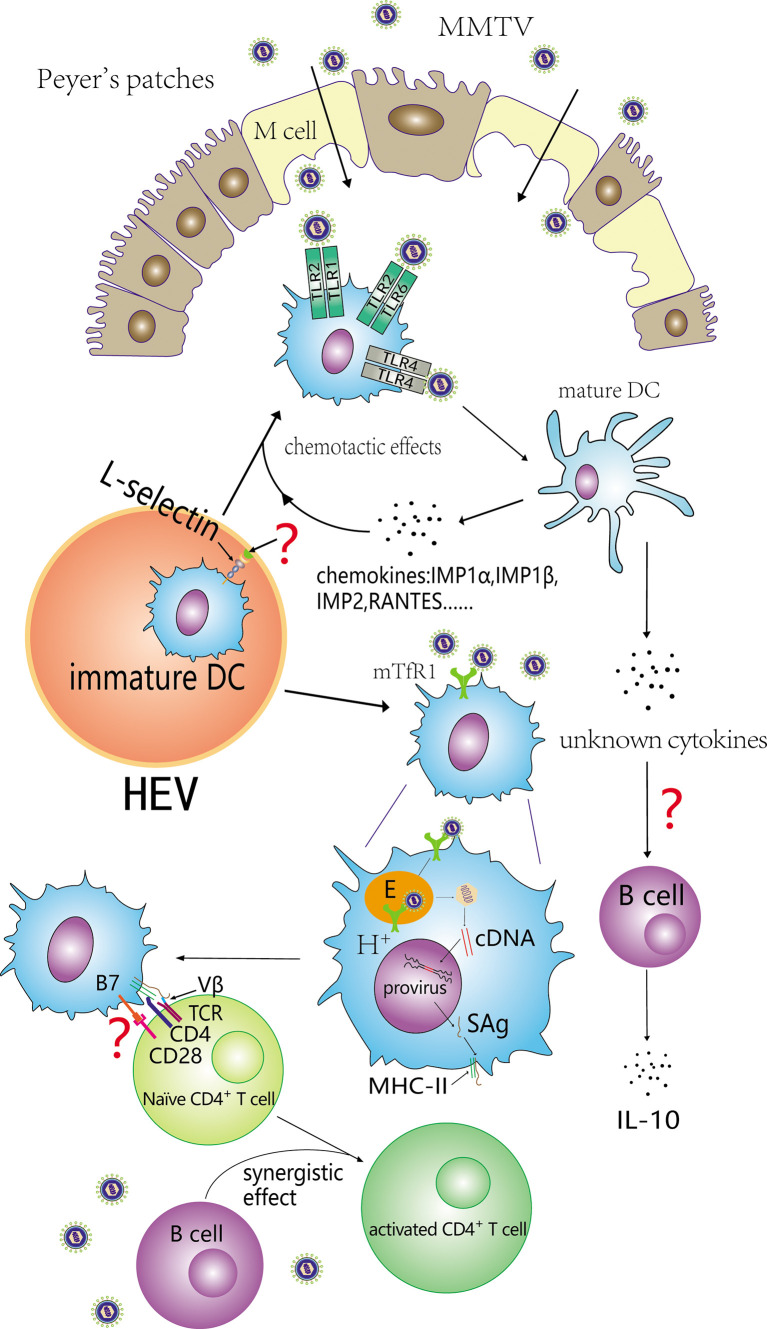
Contribution of DCs to the SAg response. Immature DCs are recruited from blood depends on L-selectin and subsequently activated by binding with MMTV. Both TLR4 and TLR2 can take part in the activation of immature DCs. Activated DCs (mature DCs) secrete some chemokines that facilitate immature DCs recruitment. In addition, some cytokines released by activated DCs can induce B cells to secrete IL-10, and inhibit the antiviral immune response. MMTV can also infect DCs and its SAg can be efficiently presented to cognate Vβ CD4^+^ T cells. DCs and B cells exert a synergistic effect on the activation of naïve CD4^+^ T cells.

In a MMTV (SW) hind footpad injection study, interdigitating DCs expressing MHCII were found in close association with proliferating Vβ6^+^ CD4^+^ T cells in the T zone of draining lymph nodes by the end of day 3. On day 5, T cell proliferation was closely associated with B220^+^ B cells located at the outer T zone instead of in interdigitating DCs, and almost all Vβ6^+^ CD4^+^ T cells were located in the T zone rather than in the medullary cords (Luther et al., 1997). We infer that virgin Vβ6^+^ T cells are initially activated by DCs from the perspective of spatial localization, which was consistent with first infection and the weak SAg response-inducing capacity observed in mixed culture (**Figure 2**).

## Lymphocytes and DCs

DCs could also be infected by MMTV (Martín et al., 2002; Vacheron et al., 2002; Burzyn et al., 2004). DCs are capable of producing infectious MMTV virions affecting various MMTV-susceptible cells including primary B cells, BMDCs, and mammary gland cells (Courreges et al., 2007) and most studies only focused on MMTV infection of B cells and T cells.

Separating lymphocyte subpopulations by FACS followed with quantitative analysis by PCR assay demonstrate that B cells play an important role for lymphocyte amplification in the SAg response (Held et al., 1993; Vacheron et al., 2002). Correspondingly, MMTV infection occurs predominantly in B cells (specifically in activated and proliferative populations of B cells) (Held et al., 1993; Held et al., 1993). T cells can also be infected by the virus and was later than B cells. Two studies based on footpad injection of MMTV (II-TES14) and MMTV (SW) showed that only B cells were infected in the draining LN on day 6 (Held et al., 1993; Upragarin et al., 1997), whereas LN samples collected on day 14 demonstrated MMTV infection in T cells (including CD8^+^ and CD4^+^ T cells, but not SAg-reactive CD4^+^ T cells) (Upragarin et al., 1997). This chronology may associate with the SAg-induced deletion of those specific Vβ-bearing T cells. The SAg response was essential for T cell infection and there were no MMTV-infected T cells in IgM knockout mice (Upragarin et al., 1997). After being infected, these T cells had the ability to delete SAg-reactive CD4^+^ T cells (Upragarin et al., 1997), played a role in carrying the virus from the gastrointestinal tract to mammary glands (via blood) and then transferred the viral activity to mammary glands (Tsubura et al., 1988). Viral expression in infected B and T cells was directed by MMTV LTR (Choi et al., 1987). Dzuris et al. (1997) demonstrated that both MMTV-infected B and T cell lines could produce MMTV virions. Essential components of a retrovirus (such as viral RNA gp52 and p27) were detected in the supernatants of these cell lines. Waander et al. (1993) reported that all B cells, CD4^+^ T cells, and CD8^+^ T cells could be infected with MMTV, and that the virus could be transmitted among these lymphocyte subsets.

## Peripheral Deletion of Cognate CD4^+^ T Cells

The gradual reduction and ultimate deletion of SAg-reactive CD4^+^ T cells in PPs was observed after a period of proliferation (Held et al., 1992; Karapetian et al., 1994), which might be due to the persistence of SAg/MHCII complexes on MMTV-infected B cells (Beutner et al., 1994). CD4^+^ T cells bearing the same Vβ TCR in other lymphoid organs including mesenteric lymph nodes, the thymus, the spleen, peripheral lymph nodes, and mammary glands, also underwent a gradual deletion (Ignatowicz et al., 1992; Tucek et al., 1993; Karapetian et al., 1994). Karapetian et al. (1994) detected MMTV (SW) cDNA in the mesenteric lymph nodes of neonates 14 days after birth and the decrease in Vβ6^+^ CD4^+^ T cells over the course of 3-4 weeks, confirming the relationship between MMTV infection and cognate CD4^+^ T cell deletion. The causal role MMTV in cognate CD4^+^ T-cell deletion of peripheral lymph nodes was also proven in a study that compared thymectomized and sham thymectomized 32-day-old mice nursed by a MMTV (C3H)-positive mother. Similar decrease kinetics for Vβ14^+^ CD4^+^ T cells in lymph nodes were observed through a kinetic line chart for sham thymectomized mice demonstrated that it was MMTV(C3H) (rather than a “dilution effect” caused by the output of newly produced Vβ14^-^ CD4^+^ T cells by the thymus) that played the primary role in inducing the peripheral deletion of Vβ14^+^ CD4^+^ T cells (Ignatowicz et al., 1992). In this study, thymocytes displayed interesting characteristics and influenced by MMTV (C3H). Both mature CD4^+^ and CD8^+^ T cells bearing Vβ14 decreased in percentage. In contrast, the percentage of immature Vβ14^+^ CD4^+^ CD8^+^ T cells remained generally unchanged. CD8^+^ T cells cannot be activated by MMTV in PPs, but Vβ14^+^ CD8^+^ T cells were unexpectedly deleted in the thymus when neonates were infected with MMTV. Peripheral Vβ14^+^ CD8^+^ T cells also underwent a gradual deletion. Unlike the peripheral deletion in Vβ14^+^ CD4^+^ T cells, Vβ14^+^ CD8^+^ T cells were unaffected by expression of the viral SAg (Ignatowicz et al., 1992).

MMTV is responsible for peripheral specific Vβ CD4^+^ T cell deletion. However, Karapetian et al. (1994) did not observe any increase in Vβ6^+^ T cells before their deletion in neonatal mesenteric lymph nodes within 3-4 weeks of birth which were nursed by a MMTV (SW)-positive mother. Similar results that MMTV (C3H) infection resulted in the deletion of Vβ14^+^ T cells were obtained in either feeding (Karapetian et al., 1994) or foot pad injection experiments. The increase of Vβ14^+^ T cells in draining lymph nodes could not be detected, and the number of Vβ14^+^ T cells decreased in peripheral blood appeared on day 4 after injection (Held et al., 1992). In contrast, the same study observed an increase in Vβ6^+^ CD4^+^ T cells in draining lymph nodes accompanied with a decrease of the same T cell subtype in peripheral blood samples when injecting mouse milk from the Vβ6^+^-deleting mother. This increase did not occur when injected with B cells from those mothers (Held et al., 1992). Vβ6^+^ CD4^+^ T cells in non-infected BALB/c mice could also undergo deletion without proliferation when injected with lymphocytes from MMTV(SW)-infected BALB/c mice (Waanders et al., 1993). Whether MMTV SAg is necessary to present to T cells or not, and the types of antigen-presenting cells, cytokines and other molecules mediating in peripheral specific Vβ T cell deletion need to study in the future. In addition, what is the reason that B cells from Vβ6^+^ deleters did not have the ability to induce proliferation of Vβ6^+^ T cells in incipient mice? Other studies about SAgs from other microbes may provide some evidences about the mechanisms of Sag inducing cognate Vβ T cell deletion. Staphylococcal enterotoxin B (SEB) was a kind of potent bacterial SAg, which can induce peripheral Vβ8^+^ T lymphocytes apoptosis (MacDonald et al., 1991; D’Adamio et al., 1993). Results of Ayroldi et al. showed the apoptotic deletion of Vβ8^+^ T cells required the co-stimulatory signal(s) from metabolically active APC by co-culturing Dcek Hi 7 cells (a fibroblast cell line expressing transfected I-E^k^ class II molecules, served as APC) with purified lymph node T cells and SEB. In addition, macrophages and DCs with metabolic activity were proven to have the ability to mediate SEB-induced Vβ8^+^ T cell deletion (Ayroldi et al., 1995). SEB-induced deletion mediated by Dcek Hi 7 cells could be significantly augmented by IL-4 and inhibited by dexamethasone (Ayroldi et al., 1995). Kuroda et al. proved that IL-2 was involved in staphylococcal enterotoxin A-induced deletion of Vβ 3^+^ and Vβ 11^+^ T cells (Kuroda et al., 1996). MMTV SAg needs to be presented by APC to cognate Vβ T cells to induce deletion. Cytokines and/or hormones may enhance or attenuate this Vβ T cell deletion.

After the interaction between SAg/MHCII complexes and cognate Vβ TCRs, specific Vβ CD4^+^ T cells are activated and, in turn, activate B cells through chemokines and direct interaction. Thus, the infected B cells proliferate and immunoglobulin is released. Since both lymphocytes and DCs can be infected by MMTV, cells in the PPs serve as a “viral reservoir” as the virus replicates (Held et al., 1993) and are therefore a source for virus spreading to other sites of the body (i.e., mammary glands) and causing disease. Chronic infection with MMTV causes a systemic deletion of cognate Vβ T cells.

## Conclusions

Although we have a certain understanding of the relationship between MMTV amplification and immune system, some details have yet to be clarified, such as what is the significance of preactivation of B cells, by which mean does MMTV integrates provirus into the genome of resting B cells, how does the virus evade immune killing effect and other issues we have mentioned in the text.

Previous studies have confirmed the existence of MMTV-like sequences in some human breast cancer (HBC) tissues (Wang et al., 1995; Etkind et al., 2000), these sequences should be exogenous since they are rarely detected in normal breast tissues whereas has a positive rate of ~30% in SBC tissues from the same individuals (Melana et al., 2001). In addition, the presence of these sequences and the tumorigenesis of HBC have been demonstrated by epidemiological studies (Wang et al., 2014; Nartey et al., 2017; Lawson and Glenn, 2019). Whether MMTV-like viruses can also be amplified by the immune response or not needs to be studied in the future.

## Author Contributions

SZhu and SZha designed the paper; contributed to manuscript writing; and approved the manuscript before submission. LC, XZ, and GL collected literatures and approved the manuscript before submission. SC and MZ gave constructive comments on the manuscript, and approved the manuscript before submission. All authors contributed to the article and approved the submitted version.

## Funding

This work was supported in part by grants from the National Science Foundation of China (#82173283 and #82103088), and Foundation of Committee on Science and Technology of Tianjin (#20JCYBJC01230). The funders had no roles in the design of the study, data collection, analysis and interpretation, or decision to write and publish the work.

## Conflict of Interest

The authors declare that the research was conducted in the absence of any commercial or financial relationships that could be construed as a potential conflict of interest.

## Publisher’s Note

All claims expressed in this article are solely those of the authors and do not necessarily represent those of their affiliated organizations, or those of the publisher, the editors and the reviewers. Any product that may be evaluated in this article, or claim that may be made by its manufacturer, is not guaranteed or endorsed by the publisher.

## Abbreviations

MMTV, mouse mammary tumor virus; Mtv, endogenous mouse mammary tumor virus; HIV, human immunodeficiency viruses; SAg, superantigen; APCs, antigen-presenting cells; PPs, Peyer’s patches; M cells, microfold cells; mTfR1, mouse transferrin receptor 1; TLR4, Toll-like receptor 4; DCs, dendritic cells; BMDCs, bone marrow-derived dendritic cells; FACS, fluorescence activated cell sorting; TCR, T cell receptor; Th2 cells, T-helper 2 cells; CS, class switching; SEB, staphylococcal enterotoxin B; LN, lymph nodes; HBC, human breast cancer.
